# Research Note: Irritating flashing light or poultry-friendly lighting—are flicker frequencies of LED luminaires a potential stress factor in the husbandry of male fattening turkeys?

**DOI:** 10.1016/j.psj.2023.103214

**Published:** 2023-10-22

**Authors:** J. Raabe, G. Raveendran, W. Otten, K. Homeyer, T. Bartels

**Affiliations:** ⁎ Friedrich-Loeffler-Institut, Institute of Animal Welfare and Animal Husbandry, Celle, Germany; † Faculty I – Electrical Engineering and Information Technology, Hochschule Hannover - University of Applied Sciences and Arts, Hannover, Germany; ‡ Research Institute for Farm Animal Biology (FBN), Institute of Behavioural Physiology, Dummerstorf, Germany

**Keywords:** fattening turkey, light-emitting diode, flicker frequency, stress, feather corticosterone

## Abstract

Conventional fluorescent tubes are increasingly being replaced with innovative light-emitting diodes (**LEDs**) for lighting poultry houses. However, little is known about whether the flicker frequencies of LED luminaires are potential stressors in poultry husbandry. The term “light flicker” describes the fluctuations in the brightness of an electrically operated light source caused by the design and/or control of the light source. In this context, the critical flicker frequency (**CFF**) characterizes the frequency at which a sequence of light flashes is perceived as continuous light. It is known that CFF in birds is higher than that in humans and that light flicker can affect behavioral patterns and stress levels in several bird species. As there is a lack of knowledge about the impact of flicker frequency on fattening turkeys, this study aimed to investigate the effects of flicker frequency on the behavior, performance, and stress response in male turkeys. In 3 trials, a total of 1,646 male day-old turkey poults of the strain B.U.T. 6 with intact beaks were reared for 20 wk in 12 barn compartments of 18 m² each. Each barn compartment was illuminated using 2 full-spectrum LED lamps. Flicker frequencies of 165 Hz, 500 Hz, and 16 kHz were set in the luminaires to illuminate the compartments. Analyses of feather corticosterone concentration were performed on fully grown third-generation primaries (P 3) of 5 turkeys from each compartment. No significant differences were found in the development of live weight, feed consumption, or prevalence of injured or killed turkeys by conspecifics reared under the above flicker frequencies. The flicker frequencies also did not significantly influence feather corticosterone concentrations in the primaries of the turkeys. In conclusion, the present results indicate that flicker frequencies of 165 Hz or higher have no detrimental effect on growth performance, injurious pecking, or endocrine stress response in male turkeys and, thus, may be suitable for use as animal-friendly lighting.

## INTRODUCTION

Light is considered a factor that can influence the behavior of poultry both positively and negatively. With the implementation of light-emitting diodes (**LEDs**) in general lighting technology, new possibilities have arisen for barn lighting. The invention of LEDs that emit wavelengths in the ultraviolet and blue spectral regions has enabled the realization of full-spectrum semiconductor-based lighting that matches the perceptual capabilities of poultry, extending into the ultraviolet range (Hart et al., 1999). However, using new lighting technologies primarily tailored to human needs raises new questions. For example, little is known about whether the technically induced effects of practical circuits are perceived as disturbing by poultry and possibly lead to performance losses or provoke undesirable behavior. Unlike incandescent lamps, LEDs cannot operate without a power supply network circuit. Typically, a driver circuit is connected upstream and contains a switching power supply that can generate different ripple currents on the load side. Unlike a thermally inert incandescent lamp, an LED directly converts these current fluctuations into luminous flux fluctuations. If the luminous flux is regulated via pulse width modulation (**PWM**) control, the current and luminous flux fluctuations are maximized. Therefore, the optical and electrical parameters of LEDs, as well as the power supply and dimming modes, should be used for evaluation.

The term light flicker describes the fluctuations in the brightness of an electrically operated light source caused by the design and/or control of the light source (IEEE, 2015). In this context, the critical flicker frequency (**CFF**) characterizes the frequency at which a sequence of light flashes is perceived as continuous light. A minimum time is required for chemical processes in the eye's retina, which are triggered during light stimulation and lead to excitation. Visual perception and sensitivity to flicker frequencies are species-specific (Inger et al., 2014). It is also important to consider the methodology used to study this sensitivity since the measurement of electroretinograms (**ERG**) results in a higher CFF than behavior-based studies of CFF (Lisney et al., 2012). In humans, the CFF is 22 to 25 Hz at low light intensities and up to 90 Hz at higher light intensities because the cones in the retina are also stimulated. However, in certain circumstances, the human eye can perceive significantly higher CFF. Furthermore, it is known that several adverse health effects occur in humans when they are exposed to invisible flickers (IEEE, 2015). As a conclusion by analogy, it must, therefore, be considered that high-frequency fluctuations in luminance, which are no longer visible to the bird but can be perceived unconsciously, can also act as stress factors in farm poultry and thus indirectly influence animal behavior. The CFF in birds is considerably higher than in humans. For example, passerine birds can resolve light-dark cycles up to 145 Hz (Boström et al., 2016). When illumination includes the UV spectrum, the temporal resolution of the visual system in birds is improved (Rubene et al., 2010). Lisney et al. (2012) examined the CFF of laying hens using ERG at different light intensities and compared them with the CFF determined in behavioral studies. They found that the values derived from the ERG were as high as 118 and 119 Hz. Thus, although chickens cannot consciously perceive flickering above 87 Hz, ERG-derived values at flicker frequencies above 100 Hz indicate an unconscious perception of light stimuli (Lisney et al., 2012). It is known from several bird species that light flicker can affect behavioral and movement patterns, the visual system, and stress levels (Inger et al., 2014). However, there is a lack of knowledge about the impact of flicker frequency on the behavior and performance of fattening turkeys. Thus, the aim of the present study was to investigate the effects of defined flicker frequencies of LED luminaires on growth performance, injurious pecking behavior, and endocrine stress response in male turkeys.

## ANIMALS, MATERIALS, AND METHODS

The husbandry conditions of the turkeys and all procedures performed in this study involving animal handling and treatments were in accordance with the German Animal Protection Law and its associated by-laws or voluntary agreements. Approval by the local ethics committee was not required as all clipped feathers were fully grown, and no innervated or vascularized tissue was removed.

### Animals, Housing, and Management

In 3 trials, a total of 1,646 male day-old turkey poults of the British United Turkeys 6 (**B.U.T. 6**) strain with intact beaks were kept for 20 wk at the Experimental Station of the Institute for Animal Welfare and Animal Husbandry (Celle, Germany). In each trial, 548 or 549 turkey poults were allocated to 12 barn compartments littered with wood shavings (floor space 18 m², with 45 or 46 turkeys per compartment (2.6 turkeys/m²), aspired stocking density at depopulation ≤53 kg/m²). Each compartment was equipped with 2 feeders (Imperator; Big Dutchman, Vechta, Germany) and 2 round drinkers (Jumbo 98; Big Dutchman, Vechta, Germany) accessible from all sides. Turkeys were fed a standard 6-phase system diet (Fa. Agravis Raiffeisen AG, Münster, Germany). Food and drinking water were provided ad libitum throughout the experiments. Weighing feeders was performed at d 14, 35, 63, 91, 119, and 140. Each barn compartment was equipped with a pecking block (Vilovoss medium; Deutsche Vilomix Tierernährung GmbH, Neuenkirchen-Vörden, Germany) for environmental enrichment. The compartments were individually climate-controlled and light-controlled, and shielded against external light. All animals were weighed on the 1st and 141st d of life. All barn compartments were visually inspected at least twice a day, usually between 7:00 am and 9:00 am and 1:00 pm and 3:00 pm. Mortality rates and causes of death, particularly related to injurious pecking, and the numbers of turkeys with fresh skin injuries caused by injurious pecking or scratching were noted also twice a day. Injured turkeys with poor prognoses were culled, whereas turkeys with minor injuries (e.g., mild scratches or superficial pecking injuries) were removed from the trial, medicated with a zinc oxide-containing skin protection spray and transferred to a separate compartment until the end of the fattening period.

### Barn Illumination

The illumination of each barn compartment was achieved using 2 full-spectrum LED lamps (Zeus LED; 30 W, λ = 350–780 nm, f = 50–16,000 Hz, Big Dutchman, Vechta, Germany). The emission spectra of the luminaires used, included both visible light (daylight, 6,500 Kelvin) and UVA spectra (i.e., full spectrum lighting). The lighting intensity was provided through 3 distinct light channels (“Sunlike,” “Lime,” and “Ultraviolet”), all modulated at a common frequency. Light was provided for 22 h on the day of arrival, with 2 intermittent dark phases from 2:00 pm to 3:00 pm and 7:00 pm to 8:00 pm. The circadian light-dark [L:D] rhythm changed from a 21 L:3 D lighting schedule on d 2 to a 16 L:8 D lighting schedule on d 7. Twilight phases of 20 min were included in the morning and evening. On the 1st and 2nd d of life, the lighting intensity was gradually reduced by 20 lx every 48 h from 100 lx to a minimum illuminance of 20 lx. This was achieved in the entire barn compartment on d 7. Lighting intensity was measured at the eye level of the turkeys using a light intensity measuring device (MAVOLUX digital, GOSSEN Foto- und Lichtmesstechnik GmbH, Nürnberg, Germany). The measuring range for illuminance was 0.1 to 199,999 lx, with error margins of maximum ±3% of the measured value for different types of light. The only variable was the flicker frequency of the luminaires.

### Flicker Frequencies

For the experimental series, pulse-width modulation was further developed and can now be adjusted on the user interface. In order to measure the flicker precisely, it was necessary to develop a measurement method that detects the light current or electrical current in the LEDs of the luminaire. A highly precise current clamp (Tektronix TCP0030A, Beaverton) was used to measure the LED current. For each of the 3 independently controllable light channels, a separate current clamp was used, making it possible to display the current flow with an oscilloscope (Tektronix MSO 2024B, Beaverton). The flicker frequencies investigated in this study were selected based on the following perspectives.

­ According to German animal protection law and its associated by-laws or voluntary agreements, the flicker frequency must be above 160 Hz when using artificial light in poultry husbandry. Health effects from invisible flicker can occur at frequencies below ∼165 Hz in humans (IEEE, 2015).
­ Davis et al. (2015) showed that humans can perceive visual flicker artifacts at frequencies up to 500 Hz.
­ At 16 kHz, a flicker frequency can be set for the luminaire used, which, according to current knowledge, can be classified as flicker-free for humans and animals.

A flicker frequency of 165 Hz, 500 Hz, and 16 kHz was set in 4 compartments per frequency. Three fattening runs were evaluated with this flicker frequency design, with the flicker frequency in the compartments changed after each trial to check the reproducibility of the results.

### Analyses of Feather Corticosterone Concentrations

To analyze feather corticosterone concentration (**fCORT**), fully grown third-generation primaries (primaries 3) on the left and right wings of 5 turkeys per compartment were clipped in the 17th wk of life within the range of the feather quill beneath the superior umbilicus using a wire cutter. Primary 3 refers to the third out of 10 outer wing feathers of a turkey (Leishman et al., 2020), and third-generation primary means that the primary has already molted twice before. In turkeys, primaries 3 are molted in the 8th wk of life (Leishman et al., 2020). All feathers were checked for integrity, particularly for physical damage to the feather vane and the presence of fault bars. Only the vaned portion (Bortolotti et al., 2008) of the most intact feathers was used for further analysis. The feathers were washed, chopped, and pulverized using a ball mill, as previously described (Bartels et al., 2021). Approximately 50 mg of feather powder was mixed with methanol (1.5 mL) and incubated for 18 to 24 h at room temperature in a slow shaker. After extraction, the samples were centrifuged at 14,000 × *g* for 5 min, and 1.2 mL of each methanol extract was aliquoted into a 1.5 mL polypropylene tube and vaporized using a vacuum evaporator. For analyses, the extract residues were reconstituted with 0.4 mL of the buffer solution of the assay kit used. Feather corticosterone concentrations were analyzed in duplicate using ELISA for corticosterone quantification (No. 501320, Cayman Chemical, Ann Arbor, MI) according to the manufacturer's instructions. All measured values were related to feather length and mass, and corticosterone concentrations were presented as pg/mm and pg/mg, respectively.

### Statistical Analyses

All statistical analyses were performed using R Studio (version 2021.09.1). For analyses, a randomized complete block design was used. The barn was divided into 2 blocks. Each flicker frequency was tested in both blocks per 2 compartments and trial, whereas the barn compartment was set as the experimental unit. Live weight at the end of the 20th wk of life; feed consumption; and fCORT data [reference “feather length”] followed normal distribution, and homogeneity of variance was confirmed using the Levene test. ANOVA for independent samples was conducted on normally distributed data to assess significant group differences. After transformation of fCORT data [reference “feather mass”], variance heterogeneity was necessitating a Welch-ANOVA. The number of injured, culled, or perished turkeys were analyzed with a nonparametric Kruskal-Wallis test. Statistical significance was set at *P* < 0.05. The results are presented as the means with standard deviation (**SD**).

## RESULTS AND DISCUSSION

After the European Union gradually banned the manufacture and marketing of incandescent lamps, which were the conventional lighting technology in poultry houses, between 2009 and 2012, they were replaced by low-pressure gas discharge tubes (fluorescent tubes). However, fluorescent and compact fluorescent lamps are also no longer available on the market in the European Union from February 25, 2023, and August 25, 2023, respectively. Therefore, LED lighting is becoming increasingly important in poultry farming. The latest developments in LED technology have made it possible to integrate illuminants as intelligent components in complex electrotechnical circuits and control them digitally. However, this new lighting technology is primarily tailored to meet human needs. Little is known about whether the flicker frequencies of LED luminaires are potential stressors in poultry husbandry.

In this study, day-old turkey poults had an average live weight of 61.8 g (SD 5.2 g). On d 141, the live weights of male turkeys kept under lights with flicker frequencies of 165 Hz and 500 Hz were 23.2 kg (SD 1.5 kg) and 23.2 kg (SD 1.4 kg), respectively, while turkeys kept under lights with a flicker frequency of 16 kHz had a body weight of 23.3 kg (SD 1.4 kg). There were no differences in live weight among the 3 experimental groups were not significant. Notably, the live weights of the turkeys in the study exceeded the performance goals of the breeding company for turkeys of the same age by approximately 8%.

Feed consumption of the turkeys was analyzed after each of the 6 feeding phases. If additional wheat grains were provided for environmental enrichment, the amount ingested was documented and summed with the regular feed intake. The feed intakes of turkeys reared under luminaires with flicker frequencies of 165 Hz, 500 Hz, and 16 kHz are shown in Table 1. The amounts of feed ingested during each feeding phase among the 3 experimental groups did not differ.

**Table 1:** Feed consumption in relation to feeding phase and luminaire flicker frequency.

Feeding phase	D	Feed consumption [kg/animal/d] (average and standard deviation [SD])		
		165 Hz	500 Hz	16 kHz
Phase 1	1–14	0.03 (SD 0.02)	0.03 (SD 0.01)	0.03 (SD 0.01)
Phase 2	15–35	0.11 (SD 0.005)	0.11 (SD 0.004)	0.11 (SD 0.004)
Phase 3	36–63	0.28 (SD 0.03)	0.27 (SD 0.02)	0.28 (SD 0.03)
Phase 4	64–91	0.46 (SD 0.04)	0.46 (SD 0.04)	0.48 (SD 0.07)
Phase 5	92–119	0.62 (SD 0.02)	0.61 (SD 0.05)	0.59 (SD 0.04)
Phase 6	120–141	0.66 (SD 0.1)	0.63 (SD 0.06)	0.67 (SD 0.09)

In compartments illuminated with luminaires with a flicker frequency of 165 Hz, 128 injured, culled, and perished turkeys were removed, whereas 97 birds were removed from those kept under luminaires with a flicker frequency of 16 kHz. In compartments illuminated with luminaires at a flicker frequency of 500 Hz, a total of 103 injured, culled, or perished turkeys were removed. No differences were observed in the rates of injured or perished male turkeys among the 3 experimental groups. Injury patterns were similar to clinical findings described elsewhere (Ferrante et al., 2019; Bartels et al., 2020). Birds injured by conspecifics exhibited deep injuries to the back of the head or neck, or both, due to skin-penetrating beak blows, some of which extended to the skull or spine.

**Figure 1:**
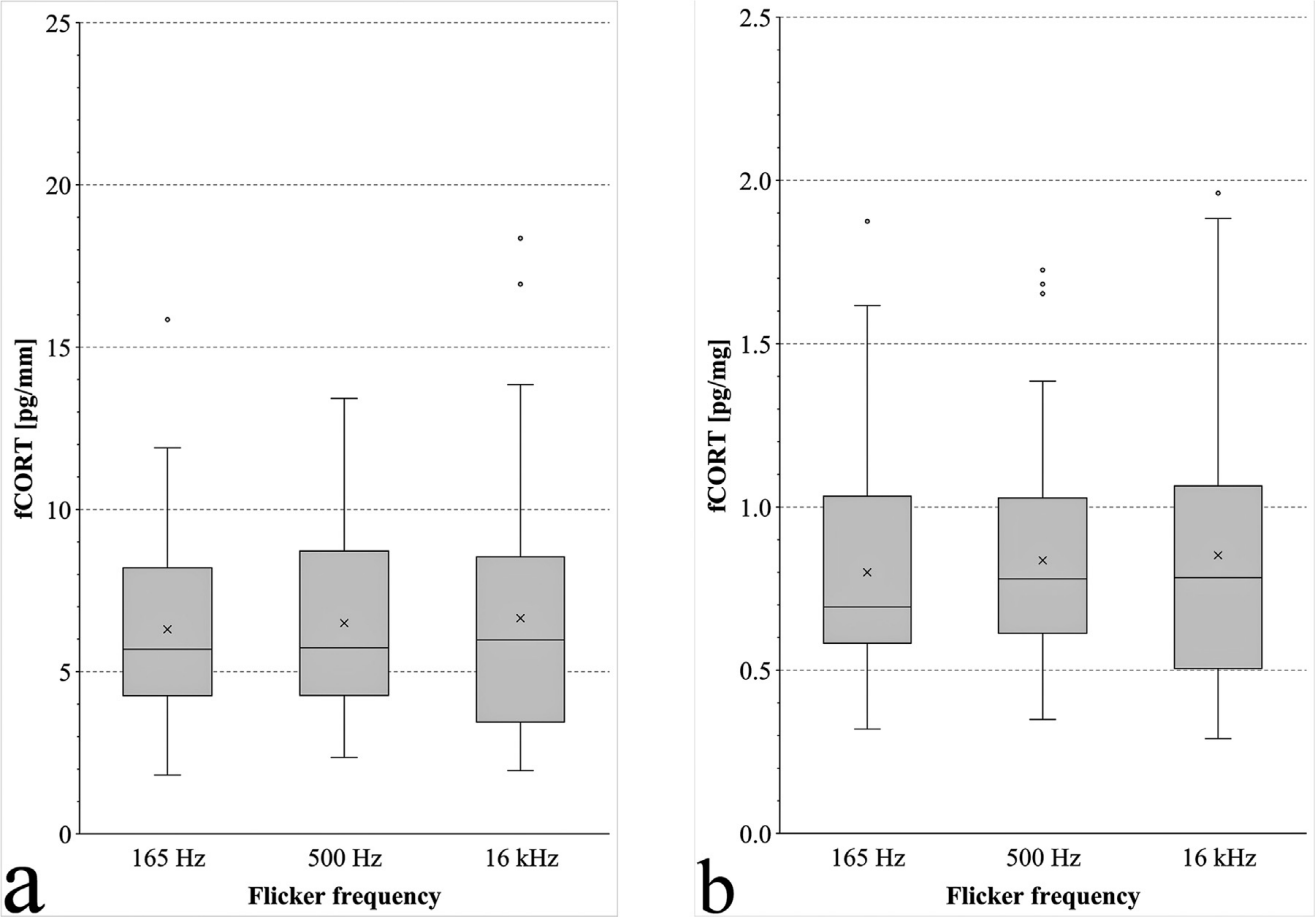
Feather corticosterone concentration in the primaries 3 of male B.U.T. 6 fattening turkeys kept under flicker frequencies of 165 Hz [*n* = 60], 500 Hz [*n* = 60], and 16 kHz [*n* = 60] in relation to feather length (A) and feather mass (B). The box and whisker plots show the minimum value, 1st quartile, median, 3rd quartile and maximum value of a data set. Box: 1st to 3rd quartile; middle line of the box: median; x: mean; whiskers: variability outside the 1st and 3rd quartiles; outlier: value that lies more than 1.5 times outside the box. There were no differences in the feather corticosterone concentrations of the primaries 3 from turkeys of the 3 experimental groups.

The measurement of fCORT is a comparatively new method for the assessment of stress in birds and may be a useful indicator in poultry welfare research (Bortolotti et al., 2008; Bartels et al., 2021). To date, the determination of stress hormones in birds has been mainly based on blood samples or excrement. However, the concentrations measured in these samples reflected comparatively short-term values. It has been known for some time that the stress hormone corticosterone is also stably stored in feathers (Bortolotti et al., 2008). Because the incorporation of corticosterone occurs during feather growth, this noninvasive method is particularly suitable for representing the long-term secretion of this stress hormone. This allowed the collection of retrospective information on stress levels over a period of several weeks without repeated sampling. To the best of our knowledge, this is the first study to describe the potential effects of artificial illumination flicker frequency on the fCORT in turkeys. The results of the fCORT analyses are shown in Figure 1A [reference “feather length”] and Figure 1B [reference “feather mass”]. When looking at the fCORT (reference “feather length”), turkeys kept under luminaires with a flicker frequency of 165 Hz showed a fCORT of 6.30 pg/mm (SD 2.67), and turkeys kept under luminaires with flicker frequencies of 500 Hz and 16 kHz had a fCORT of 6.50 pg/mm (SD 2.69) and 6.64 pg/mm (SD 3.65), respectively. The differences between the fCORTs of the experimental groups were not significant. A comparison of the fCORT [reference “feather mass”] (Figure 1B) for turkeys kept in compartments illuminated by luminaires with a flicker frequency of 165 Hz revealed a fCORT of 0.80 pg/mg (SD 0.29), while those kept under luminaires with flicker frequencies of 500 Hz and 16 kHz showed fCORTs of 0.84 pg/mg (SD 0.31) and 0.85 pg/mg (SD 0.42), respectively. In accordance to fCORTs [reference “feather length”], the differences between fCORTs (reference “feather mass”) were not significant. Stress can be associated with reduced performance in fattening poultry because glucocorticoids affect protein metabolism with a decrease in protein synthesis and an increase in skeletal muscle degradation (Scanes, 2016), consistent with findings that the live weight of the turkeys was not affected by the selected flicker frequencies up to 20 wk of life.

In summary, our results indicated that no statistically significant differences were found in male nonbeak treated fattening turkeys under artificial light with flicker frequencies of 165 Hz, 500 Hz, and 16 kHz with respect to live weight development, feed consumption, and prevalence of injured turkeys or turkeys killed by conspecifics. Flicker frequencies also had no effect on feather corticosterone concentrations in turkey primaries.

The present results allowed us to conclude that flicker frequencies of 165 Hz or higher have no detrimental effects on the live weight development of turkeys. The frequency of injurious pecking was neither positively nor negatively influenced by the flicker frequencies examined. In addition, fCORT, as an indicator of long-term stress, was neither reduced nor increased at the selected flicker frequencies. In this respect, our investigations indicate that luminaires with flicker frequencies of 165 Hz or higher meet the requirements of animal welfare in Germany and are suitable for lighting fattening turkey houses if they emit a light spectrum adapted to the perception of turkeys.
